# Investigating the power of eyes open resting state EEG for assisting in dementia diagnosis

**DOI:** 10.1186/s13195-022-01046-z

**Published:** 2022-08-05

**Authors:** Jack L. Jennings, Luis R. Peraza, Mark Baker, Kai Alter, John-Paul Taylor, Roman Bauer

**Affiliations:** 1 School of Computing, Newcastle University, Newcastle upon Tyne, UK; 2 Biosciences Institute, Newcastle University, Newcastle upon Tyne, UK; 3 IXICO Plc, London, UK; 4 Translational and Clinical Research Institute, Faculty of Medical Sciences, Newcastle University, Campus of Ageing and Vitality, Newcastle upon Tyne, NE4 5PL UK; 5 Department of Clinical Neurophysiology, Royal Victoria Infirmary, Queen Victoria Rd, Newcastle upon Tyne, NE1 4LP UK; 6 Faculty of Medical Sciences, Newcastle University, Framlington Place, Newcastle upon Tyne, NE2 4HH UK; 7 Department of Computer Science, University of Surrey, Guildford, GU2 7XH UK

**Keywords:** Electrocenphalography, Eyes open, Eyes closed, Quantitative, Machine learning, Dominant frequency, Alzheimer’s disease, Parkinson’s disease, Lewy body dementia

## Abstract

**Introduction:**

The differentiation of Lewy body dementia from other common dementia types clinically is difficult, with a considerable number of cases only being found post-mortem. Consequently, there is a clear need for inexpensive and accurate diagnostic approaches for clinical use. Electroencephalography (EEG) is one potential candidate due to its relatively low cost and non-invasive nature. Previous studies examining the use of EEG as a dementia diagnostic have focussed on the eyes closed (EC) resting state; however, eyes open (EO) EEG may also be a useful adjunct to quantitative analysis due to clinical availability.

**Methods:**

We extracted spectral properties from EEG signals recorded under research study protocols (1024 Hz sampling rate, 10:5 EEG layout). The data stems from a total of 40 dementia patients with an average age of 74.42, 75.81 and 73.88 years for Alzheimer’s disease (AD), dementia with Lewy bodies (DLB) and Parkinson’s disease dementia (PDD), respectively, and 15 healthy controls (HC) with an average age of 76.93 years. We utilised k-nearest neighbour, support vector machine and logistic regression machine learning to differentiate between groups utilising spectral data from the delta, theta, high theta, alpha and beta EEG bands.

**Results:**

We found that the combination of EC and EO resting state EEG data significantly increased inter-group classification accuracy compared to methods not using EO data. Secondly, we observed a distinct increase in the dominant frequency variance for HC between the EO and EC state, which was not observed within any dementia subgroup. For inter-group classification, we achieved a specificity of 0.87 and sensitivity of 0.92 for HC vs dementia classification and 0.75 specificity and 0.91 sensitivity for AD vs DLB classification, with a k-nearest neighbour machine learning model which outperformed other machine learning methods.

**Conclusions:**

The findings of our study indicate that the combination of both EC and EO quantitative EEG features improves overall classification accuracy when classifying dementia types in older age adults. In addition, we demonstrate that healthy controls display a definite change in dominant frequency variance between the EC and EO state. In future, a validation cohort should be utilised to further solidify these findings.

**Supplementary Information:**

The online version contains supplementary material available at 10.1186/s13195-022-01046-z.

## Introduction

As of 2018, dementia has reached an estimated prevalence of 50 million people worldwide with an expected increase to 75 million cases by 2030 [1]. Dementia with Lewy body (DLB) pathologies being found in 25–45% of dementia cases post-mortem are likely showing an under-representation in current data [2, 3]. Notably, most cases of DLB are misdiagnosed as the more common Alzheimer’s disease (AD) [4].

Alzheimer’s disease currently represents the largest proportion of dementia cases globally [5] and is characterised by an irregular build-up of the polypeptide beta amyloid, which forms plaques within brain regions such as the hippocampus leading to the damage of neurons [6]. Another common group is characterised by the Lewy body disease (LBD) pathology, being comprised of Parkinson’s disease dementia (PDD) and DLB with a common pathology of Lewy body protein building up within specific brain regions [5, 7].

The initial overlap symptomatically of some dementia types is problematic, particularly due to the differential treatment required. For example, DLB is often misdiagnosed as the more common AD due to presenting similar symptoms early in disease progression such as memory loss and language pathology [4, 8]. Those with DLB are however much more sensitive to commonly prescribed neuroleptics and require more specific and targeted treatment [9]. DLB also has symptomatic overlap in later disease progression stages with the dementia type PDD. This manifests with physical tremors, problems with balance and a shuffling gait in addition to memory loss; differentiation between these two diseases currently is highly subjective as it normally relates to which symptoms manifest first. If cognitive impairment develops first, a patient will often be diagnosed with DLB; however, if tremors develop first, a Parkinson’s diagnosis is usually given. New research though has shown the presence of mild cognitive impairment (MCI) in Parkinson’s disease patients (PD) with no current dementia diagnosis [10], which presents a possible further blurring of the line between PDD and DLB. Multiple other similarities displayed between the PDD and DLB neuropathologically compound the belief that these diseases represent regions of a larger spectrum. Commonly, both are grouped into the Lewy body dementia group [11]. However, despite similarities, the more aggressive disease progression of DLB leads to a shorter life expectancy, likely necessitating the development of a differential treatment between the two LBD dementias in the future.

Electroencephalography (EEG) is a tool for the detection of biomarkers related to surface brain activity and is often used in the clinical diagnosis of other neurological conditions such as epilepsy [12]. EEG records brain activity through use of nodes placed across the scalp in specific locations. The brain activity is recorded in the form of spectral data. EEG node density can vary based upon the specific layout which is quantified via distance. The 10-5 system which was utilised in this study comprises nodes that are densely packed on the scalp. Data can then be interpreted as a visual spectrum of activity through which quantitative results can be gathered, often referred to as quantitative EEG (qEEG). Some studies have already shown promise for the ability of qEEG to classify between different dementia types [13–15]. Importantly, clinical EEG is a non-invasive and cost-effective data acquisition technique at around $300 USD [16] and is already widely available. Clinical EEG systems have a significantly lower cost than current gold standards such as brain MRI or DatScan, with respective costs of $600 USD and $1200 USD each [16] (all prices are based on UK private patient healthcare costs as of 2015). Techniques such as DatScan also require small dosing with radioactive tracers which limits repeat data acquisition in addition to limited global availability.

Currently, EEG is not used for the diagnosis of dementia as a whole and is instead primarily an interest of research; however, EEG has become popular as an auxiliary tool for the clinical diagnosis of DLB patients displaying its utility as a diagnostic tool [17]. The possibility of further expanding EEG as an auxiliary biomarker detection method in addition to its overall affordability would allow for more frequent screening which could assist in targeted treatment for patients on an individual basis.

As a tool for assisting in dementia research, EEG is most often recorded in an eyes closed resting state (EC) where participants remain awake while performing no task or movements. It has been shown that, during EC resting state EEG, dementia patients display a definite decrease in power for the alpha frequency range within the EEG spectrum when compared to healthy participants [18, 19].

A similar procedure can be performed with eyes kept open (EO), commonly used for the diagnosis of conditions such as epilepsy or other seizure-related neurological conditions. In this case, participants are asked by their clinician to open and close their eyes [20]. This is currently not widely utilised for dementia research despite the routine EO EEG data acquisition in clinical neurophysiology, as during EO EEG the alpha peak of dementia patients does not display the same significant decrease when compared to healthy participants. Recently, however, a significant impairment has been found within the EO resting state alpha reactivity of LBD patients when compared to those with AD, thus representing the need for further investigation into the EO dataset [21].

Previous studies that utilised qEEG features as a tool for the classification of AD, DLB and PDD dementia groups have focussed on the EC resting state EEG of patients [13–15]. In this study, we investigate the hypothesis that the inclusion of EO resting state EEG data represents a valuable source of statistically significant qEEG features, improving classification accuracies in dementia diagnosis.

## Methods

The dataset used in this study is the same as that used by Peraza et al. [13]; hence, the assessments used for participants as well as the process of cleaning EEG recordings and data acquisition are the same.

### Participant assessment

In total, 98 participants were recruited from the North East Region of England. Those with dementia were recruited from a population that had been referred to old age neurology services and clinics. In total, 80 dementia patients were recruited for the study; this included 32 AD patients (22 male, 10 female), 26 DLB patients (21 male, 5 female) and 22 PDD patients (20 male, 2 female). Along with these dementia patients, 18 age-matched healthy controls (11 male, 7 female) were also recruited for between-group comparisons [13]. A diagnosis of dementia was made by two experienced old age psychiatrists who followed the clinical diagnosis criteria for DLB [4, 22], the diagnostic criteria for PDD [23] and the AD National institute on Aging-Alzheimer’s Association criteria for the diagnosis of AD [8].

For this study, participants underwent in-depth neurological and neuropsychiatric testing. The Cambridge Cognition Examination (CAMCOG) and Mini-Mental state exam (MMSE) were used to assess cognitive function in patients, with both tests being commonly used for assessing the extent of a participant’s dementia symptoms. Additionally, the Neuropsychiatric inventory test was performed to assess the severity and frequency of hallucinations for participants (NPI hal).

A breakdown of the demographics and clinical variables for the participant cohort in this investigation is given in Supplementary Table 2. No significant differences were observed between groups for both age and gender. Similar overall global cognitive impairment was displayed between the dementia groups. However, the AD group displayed significant differences with the DLB and PDD groups in lower CAMCOG memory impairment and NPI hallucinations when utilising a Bonferroni correction (*p* < 0.05), which is displayed in supplementary Table 3. Additionally, it was found that AD patients displayed a significant difference in CAMCOG total score when compared to DLB patients which is not seen when comparing the AD and PDD patients, which can also be found in supplementary Table 3.

Importantly, there were no significant differences in the usage of cholinesterase inhibitors across dementia groups. The Bonferroni-corrected values are displayed in supplementary Table 4.

### Electroencephalography and signal processing

In total, 150 s of resting state EEG was acquired using a 128 sintered Ag/AgCl electrode Waveguard cap (ANT Neuro, The Netherlands) placed in a 10-5 positioning system [24] for each participant. Channels were recorded at a sample rate of 1024 Hz with an electrode impedance of no more than 5kΩ [13]. Before the analysis of qEEG features, all EEG pre-processing and cleaning was carried out blinded to group membership using EEGLAB [25] MATLAB functions (R2012; MathWorks, Natick Massachusetts) for both EC and EO recordings [13]. After pre-processing and cleaning, the following 7 processing steps were conducted: (1) Baseline components were subtracted from EEG channels and a phase invariant band pass was applied between 0.3 and 54 Hz with a 2nd order Butterworth filter. (2) Bad channels were deleted from EEG recordings based on noise, bad contact or zero amplitude, and the Fz reference channel was also deleted for each EEG recording. (3) The full EEG recording was inspected for noisy time-bound artefacts that contaminated all channels such as chewing and swallowing. These bad segments were then deleted, and the EO state required the deletion of additional segments due to greater noise compared to the EC state. (4) EEG with ICA implemented using fast ICA [26] through EEGLAB default parameters and used to reduce noise via the removal of eye artefacts, and the 50 Hz power line component and heartbeats were identified and deleted, with no more than 12 artefactual independent components being deleted. (5) After the initial use of ICA, some EEG recordings still showed artefacts and so ICA was repeated, and no more than 12 ICs were deleted. (6) Previously deleted channels were then interpolated back using information from surrounding electrodes using spherical interpolation in EEGLAB. (7) EEG data was then referenced to the average reference using EEGLAB [13] with recordings being segmented into 2-s windows with a 1-s overlap.

From the original 98 subjects, 65 were used for analysis, including 15 HC, 12 AD, 21 DLB and 17 PDD subjects. The other 33 patients (2 HC, 13 AD, 4 DLB, 4 PDD) were removed from the dataset due to participants not having at least 20 s of combined resting state eyes closed or eyes open EEG after cleaning [13]. This criterion was employed because 20 s of continuous resting state EEG has been shown to be the required amount to account for the inherent variability in EEG [27].

### Spectral analysis

EEG segments were analysed over 5 cortical regions: frontal (F), central (C), temporal (T), parietal (P) and occipital (O). Within each of these regions, the relative power for the delta (0.5–4 Hz), theta (4–5.5 Hz), high theta (5.5–8Hz), alpha (8–13 Hz) and beta (13–30 Hz) frequency bands in addition to each subject’s dominant frequency (DF) and dominant frequency variance (DFV) were calculated using a Welch’s Periodogram; 2048 sample segments, using a Hamming window with a 50% overlap between segments as well as a fast Fourier transform size of 2^13; giving a frequency resolution of 0.125 Hz. Here, DF is defined as the power spectrum with the highest power frequency bin between 4 and 15 Hz with an average taken across the time series frequency bins for the mean, and DFV is the standard deviation across these segments [13]. Relative power spectral density was used as to account for inter-patient variability that is present in non-normalised data, such as neurophysiology and tissue properties [13, 18, 28].

### Statistics

The statistical analysis of qEEG features was conducted using the MATLAB statistical toolbox feature (R2018a; MathWorks, Natick Massachusetts), WEKA [29] and SPSS (SPSS Statistics for Windows, version 23.0. IBM). qEEG measures were analysed using a one-way ANOVA for the four groups using a post hoc unpaired Bonferroni *t*-test for between-group comparison, and any features found not to have normally distributed data were assessed similarly using the Kruskal-Wallis non-parametric test. Normality of data was tested for by quartile-quartile plots. A *p* value of less than 0.05 was considered statistically significant (*p* < 0.05), and the significance value of the post hoc tests was automatically corrected so that a *p* value of 0.05 represented a statistically significant difference between group means.

### Feature selection

**Fig. 1:**
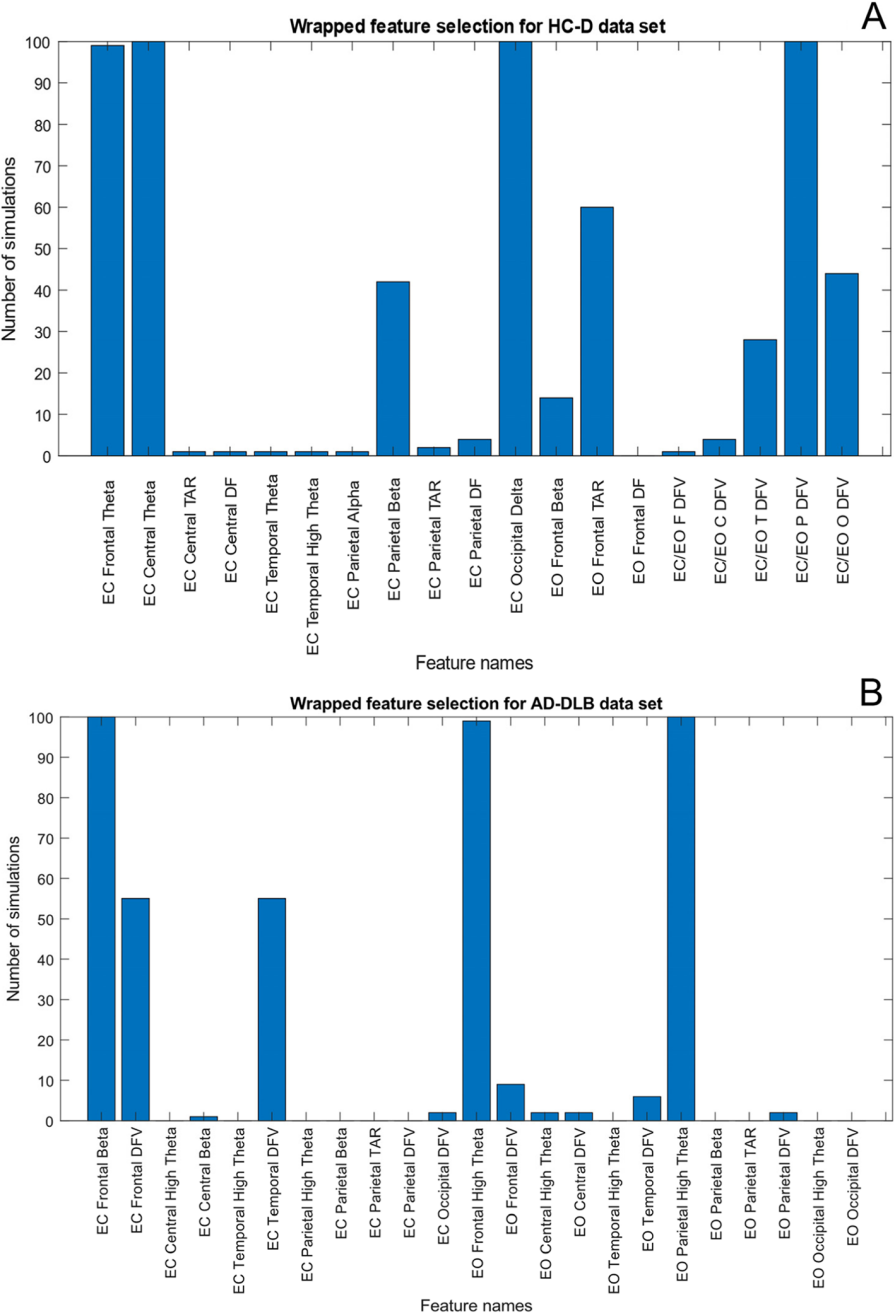
Figures showing the total number of times that features for HC-D (**A**) and AD-DLB (**B**) classification. Wrapped feature selection utilised training and testing datasets and was simulated 100 times such that model consistency could be ascertained. In both cases, several features were selected consistently, with other features which were selected less adding redundant information that did not improve classification accuracy. With features consisting of the relative delta, theta, high theta, alpha and delta power in addition to the ration of the high theta-alpha relative power (TAR) dominant frequency (DF), dominant frequency variance (DFV) and the ratio of the dominant frequency variance between the EC and EO state (EC/EO)

Feature selection is critical to the reliability of machine learning algorithms as it prevents the overfitting of data while removing redundant features, such that the overall generalisability of the created classifier is maximised. Feature selection was performed in WEKA [29] and MatLab (R2018a; MathWorks, Natick Massachusetts) using neighbourhood component analysis (NCA). NCA utilises a gradient ascent technique as well as a leave-one-out cross-validation to maximise the classification accuracy and to remove redundant features from the dataset [30]. For feature selection, participant data was split into training and testing to avoid overestimation of cross-validation accuracy. This feature selection method produced a weight for each feature allowing it to be ranked against all other features for future classification as well as removal of redundant features. We performed wrapper style feature selection, utilising 100 simulated runs to ascertain the consistency of our feature selection method. Wrapped feature selection provides as subset of provided features that improve classification accuracy without adding redundant information. Two bar charts displaying the number of times features improved classification accuracy can be found in Fig. 1A and B, for differentiation between HC vs dementia groups and AD vs DLB groups, respectively.

### Classification and machine learning algorithms

Several supervised machine learning methods were evaluated using the k-fold cross-validation (CV) method in MATLAB and WEKA [29] for HC-dementia, AD-DLB and DLB-PDD classification.

Selected features were used to train machine learning classifiers utilising 10-fold cross-validation. The k-nearest neighbour model was employed for classification between different patient groups. Moreover, both logistic regression and support vector machines were used to assess the impact of the EO dataset on classification accuracy. For each classifier, a receiver operating curve (ROC) was also examined to determine the area under the curve value (AUC) and the confidence interval (CI) of classification results. Additionally, logistic regression and support vector machine models were used to compare the effect that inclusion of EO data had on overall classification accuracy; these machine learning methods are compared due to their common use in dementia classification based on EEG data.

Finally, we have used a multi-class k-nearest neighbour machine learning approach to filter HCs from non-healthy participants (step 1) and then classify each dementia group individually against other individuals determined to be non-healthy (step 2). This is done so that each of the 4 classes is classified within the same setting. This can be seen as emulating how machine learning is likely to assist in clinical setting. For non-healthy individuals, the classifier (step 2) determines whether the patient is one of the three dementia subgroups (AD, DLB and PDD). As above, we have performed this classification technique both with and without the addition of EO data alongside EC data to determine what if any benefit the addition of EO data has on classification results.

## Results

### Neighbourhood component analysis (NCA)

NCA was performed on all datasets to remove redundant features (that could lead to decreased classification accuracies for machine learning due to overfitting). Notably, NCA transforms and maximises the performance of features for utilisation in k-nearest neighbour [31]. We performed these simulation 100 times to ascertain model consistency, and as can be seen in Fig. 1A and B, the overall model consistently chooses the same features for classification across multiple runs, with 4 features chosen in >95% of runs for HC-dementia and 3 features for AD-DLB. For HC vs dementia, the EC frontal high theta, EC central theta, EC occipital delta and the ratio between the EC and EO dominant frequency variance (DFV) in the parietal region were chosen; for AD-DLB, EC frontal beta, EO frontal high theta and EO parietal high theta; for AD-DLB/PDD, the EC temporal DFV, EO frontal high theta, EO parietal high theta, EO occipital high theta and EO occipital DFV; and finally for DLB-PDD classification, only the EC parietal alpha and EO occipital delta features were chosen. Features were utilised for machine learning if they were picked in 95% of simulated runs; a full list of features tested for the HC-D group is displayed in supplementary Fig. 1.

### Quantitative EEG and dominant frequency analysis

From the one-way four group ANOVA, it was found that there were significant differences for the eyes closed DF between the HC and dementia groups in the parietal and occipital regions, with dementia groups displaying a mean slowing in their DF towards the high theta frequency range. Additionally, we found the same significant difference between the HC and dementia patients DF in the EO resting state within the same regions. These results are displayed in supplementary Table 5.

Similarly, we investigated the significance of DFV in the EC and EO state between groups. By computing the ratio between the two states, we found that healthy controls displayed a significant decrease in variance in the EC compared to EO state. Notably, this change was found to be significant across all cortical regions for the healthy control groups when compared to dementia groups. For the AD, DLB and PDD groups, we found no significant change in DFV between the EC and EO states. These results are displayed in supplementary Table 6. Additionally, we investigated the EEG data scroll of participants between the two states and found that HC participants displayed an apparent change in frequency that was not seen for dementia patients, with some exemplary examples shown in supplementary Fig. 2.

The ratio of the theta and alpha relative power (TAR) also showed a significant difference between the HC and dementia groups within the occipital region in both the EC and EO resting states. The parietal region showed a significant difference between the HC and the dementia groups within the EC state. However, in the EO state, only the DLB and PDD groups were found to have a significantly greater TAR than the HC groups.

### Inter-group classifications

We investigated inter-group group separability using machine learning classification for EC and EO spectral features. To this end, we only used features which had been chosen via feature selection. A k-nearest neighbour model classifier was chosen for inter-group classification. A *k* value of 10 was selected for cross fold validation between different participant groups. A summary of the results for classification between healthy controls and dementia patients in addition to classification between the AD, DLB and PDD groups can be found in Table 1 for EC and EO qEEG features.

**Table 1:** Comparison of classification results between HCs and dementia patients as well as AD and DLB using 10-fold cross-validation. Utilising a k-nearest neighbour machine learning model, in addition, we present the confidence interval (CI) for the specificity and sensitivity of each classification type

Machine Learning classification accuracies				
Classification Type	Accuracy	Specificity (± CI)	Sensitivity (± CI)	Weighted Average AUC
HC-D	0.91 ± 0.07	0.87 ± 0.09	0.92 ± 0.08	0.85
AD-LBD	0.86 ± 0.10	0.75 ± 0.19	0.9 ± 0.13	0.76
AD-DLB	0.82 ± 0.13	0.75 ± 0.19	0.81 ± 0.17	0.74
DLB-PDD	0.61 ± 0.16	0.76 ± 0.20	0.3 ± 0.22	0.61

Table 2 summarises the classification results between the AD and DLB patient groups when utilising only EC qEEG features and combining EC and EO qEEG features, for the k-nearest neighbour algorithm, logistic regression and a quadratic support vector machine.

**Table 2:** AD-DLB classification result comparison for EC and EC-EO classification using 10-fold cross-validation, with comparisons across 3 separate machine learning models: k-nearest neighbour, logistic regression and support vector machine

AD-DLB classification accuracies				
Classifier	Accuracy	Specificity (± CI)	Sensitivity (± CI)	Weighted Average AUC
EC + EO data				
Cosine KNN	0.82 ± 0.13	0.75 ± 0.19	0.81 ± 0.17	0.74
Logistic Regression	0.76 ± 0.15	0.67 ± 0.20	0.86 ± 0.15	0.84
Quadratic SVM	0.82 ± 0.13	0.58 ± 0.21	0.95 ± 0.09	0.82
EC data only				
Cosine KNN	0.67 ± 0.16	0.5 ± 0.21	0.76 ± 0.18	0.63
Logistic Regression	0.73 ± 0.15	0.58 ± 0.21	0.81 ± 0.17	0.77
Quadratic SVM	0.73 ± 0.15	0.58 ± 0.21	0.81 ± 0.17	0.72

Additionally, we investigated the change in classification accuracy for AD vs DLB classification when one includes clinical scores, with CAMCOG memory score being chosen during feature selection alongside EC frontal beta and EO parietal high theta, with a marked increase in accuracy for identifying patients with DLB. These results are presented in Table 3.

**Table 3:** Improved AD-DLB classification results when including CAMCOG memory total score for classification, done for both EC + EO and EC only classification

AD-DLB classifications with CAMCOG memory inclusion				
Data	Accuracy	Specificity	Sensitivity	Weighted Average AUC
EC + EO	0.88 ± 0.12	0.75 ± 0.19	0.95 ± 0.09	0.94
EC	0.73 ± 0.27	0.67 ± 0.20	0.76 ± 0.18	0.71

### Multi-class classification results

Finally, we have investigated the capability of a multi-class classification approach to differentiate HCs from non-healthy participants and then differentiate each dementia type from other participants determined to be non-healthy. Again, we investigated if the addition of EO data improves classification results. These results are displayed in Table 4.

**Table 4:** Comparison of two step classification results using EC and EC-EO data for classification with 10-fold cross-validation. Classification was done using the k-nearest neighbour. Each classifier first identifies and filters HCs from non-healthy participants (part 1), and those identified as non-healthy are then carried through to the next stage of classification. Non-healthy individuals (part 2) are then classified within the multi-class environment based on which of the three dementia subgroups they are likely to belong to (AD, DLB, PDD)

	**Classifier 1 (EC+EO)**					
	**Accuracy**	**Specificity**	**CI Spec**	**Sensitivity**	**CI Sens**	**Weighted Average AUC**
	Step-1 HC against Non healthy					
HC	87.70 ± 1.34	86.0 ± 7.3	0.10	93.3 ± 1.7	0.13	0.91 ± 0.04
Dementia Type	Step 2 - Separation of individual dementia groups					
AD	83.20 ± 1.10	89.47 ± 3.71	0.10	75.00 ± 0.76	0.28	0.83 ± 0.01
DLB	85.60 ± 0.89	88.89 ± 1.48	0.13	90.48 ± 6.23	0.13	0.86 ± 0.01
PDD	96.8 ± 1.10	90.09 ± 9.3	0.00	88.24 ± 2.64	0.16	0.98 ± 0.03
	**Classifier 2 (EC Only)**					
	**Accuracy**	**Specificity**	**CI Spec**	**Sensitivity**	**CI Sens**	**Weighted Average AUC**
	Step-1 HC against Non healthy					
HC	84.30 ± 2.29	73.30 ± 2.00	0.13	86.00 ± 5.57	0.18	0.88 ± 0.04
Dementia Type	Step 2 - Separation of individual dementia groups					
AD	81.60 ± 4.98	84.22 ± 7.46	0.13	58.3 ± 2.94	0.37	0.73 ± 0.04
DLB	79.2 ± 1.79	81.46 ± 5.21	0.16	81.00 ± 3.53	0.19	0.86 ± 0.03
PDD	96.4 ± 4.98	90.09 ± 9.3	0.00	88.2 ± 0.89	0.16	0.98 ± 0.03

## Discussion

Through spectral data analysis, it was found that dementia participants, within our study, displayed a definite mean EEG slowing between 4 and 13 Hz. This slowing is most prominently seen as a decrease in dominant frequency in the occipital and parietal brain regions when comparing between healthy controls and dementia patients. This decrease in DF can be most prominently seen in the DLB and PDD groups, with the theta-alpha relative power ratio of both groups also being significantly different from the healthy control group for both regions. These findings are in agreement with already well-documented literature on EC EEG slowing [13, 14, 18, 19]. Additionally, our results suggest that there is also a significant decrease in the dominant frequency of dementia patients when compared to healthy controls in the occipital and parietal regions in the EO state, and this data is presented in supplementary Table 5. We also must consider however that the inherent variance in dementia conditions may require greater statistical rigour. Thus, if the significance level is decreased to *p* ≤ 0.01, the significant change in dominant frequency for the EO state is only present between the HC and DLB+PDD groups. A larger independent validation cohort is required to strengthen this finding and to help minimise inherent variance in the dementia groups and validate the AD findings.

**Fig. 2:**
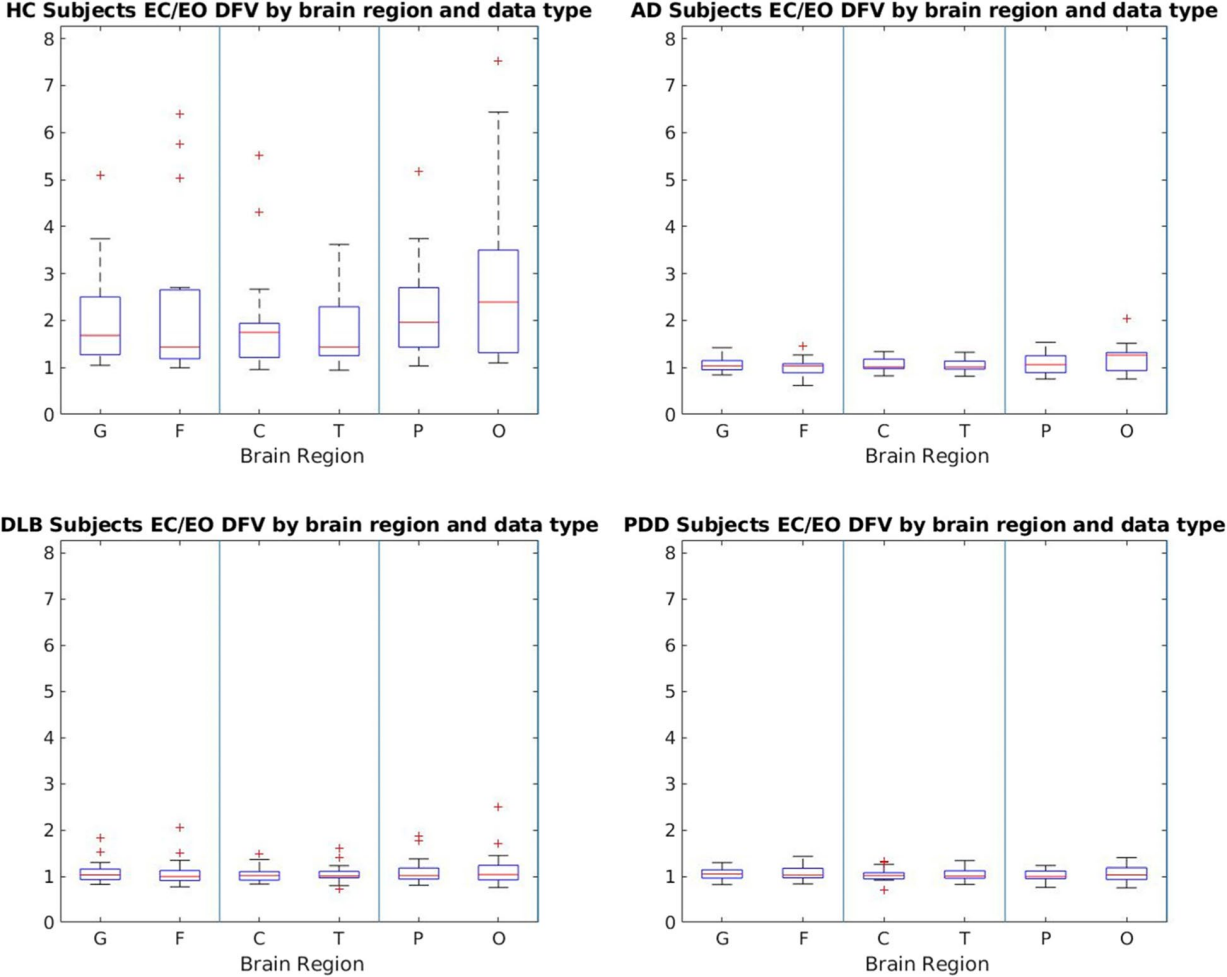
Box plots for the dominant frequency variance (DFV) ratio in the eyes closed (EC) and eyes open (EO) resting state for HC, AD, DLB and PDD participants across all cortical regions. These boxplots display a possible difference between healthy and dementia participants when comparing eyes closed and open states that has yet been uncommented upon in literature for inter-group differentiation and may be representative on an underlying biomarker. The HC group showed a significant difference (*p* < 0.05) in comparison to all dementia groups for the ratio of EC and EO DFV. In addition, no dementia group was found to have a significant difference with any group other than HC, as shown in supplementary Table 6. “–” is the median DFV value of the group, with “+” representing outliers

In healthy controls, we found a significant increase in dominant frequency variance (DFV) in the EC state compared to the EO state as shown in Fig. 2. This difference in DFV between EC and EO was not seen in any dementia group. This difference was yet undocumented in relevant literature. It is possible that there may be further features within the EO dataset that have yet to be investigated. We display the EEG data scrolls in the occipital regions for a HC, AD, DLB and PDD participant in supplementary Fig. 2, with the occipital region being chosen as not only do we see the greatest difference in DFV between states for HC participants in this region, but these nodes also display prominent alpha rhythms for HC participants. We found that the HC participant displayed a decrease in wavelength in the EO state when compared to the EC state, which is not the case in the AD, DLB or PDD participants. This may indicate a reason for the change in DFV of HC participants. It is notable that despite the large variance in all three dementia conditions the difference between the HC and dementia groups was still found to be significant up to *p* ≤ 0.01. For both the DLB and PDD groups, this significance was found to a value of *p* ≤ 0.001. These statistics are displayed in supplementary Table 6.

For our first inter-group comparison, we investigated the classification between HC and dementia participants when utilising features selected through feature reduction. Utilising the k-nearest neighbour (KNN) machine learning method, we achieved a specificity of 0.87 and sensitivity of 0.86. Good classification accuracies were achieved, in line with classification results being comparable with those of Peraza et al. [13] who utilised more complex network analysis EEG features.

Secondly, we investigated the AD-DLB classification type, achieving results of 0.75 and 0.86 for sensitivity and specificity, respectively. These classification results are similar to those of Zande et al. [14] who achieved a specificity of 0.87 and sensitivity of 0.83 and Pascarelli et al. who achieved a diagnostic result of 0.75 sensitivity and 0.85 specificity [32] or Mehraram et al. who achieved 0.47 sensitivity and 1.00 specificity [33]. Unlike our investigation, other studies combined either the EC resting state qEEG of participants with network-derived connectivity measures [14] or investigated inter-group differences using EEG cortical sources which are more difficult to acquire in clinical settings [32], without the inclusion of EO qEEG data. Notably, our results for AD-DLB classification are comparable to FP-CIT-SPECT, which currently acts as the gold standard in diagnosis of dementia patients [34–36]. However, unlike EEG, FP-CIT-SPECT scans are limited in repeat data acquisition due to requiring the use of single gamma photon emission for imaging. Additionally, we investigated AD vs DLB/PDD classification, which achieved a specificity of 0.75 and a sensitivity of 0.90 (with all PDD patients being successfully differentiated from those with AD). Finally, we investigated the classification accuracy between the DLB and PDD dementia groups with a specificity of 0.76 and a sensitivity of 0.30. The ability to achieve such classification results between AD and DLB through the utilisation of only qEEG features from the EC and EO EEG dataset is highly applicable to assisting in dementia classification in a clinical environment due to the relative simplicity of data acquisition and interpolation without the need for a more complex method. Finally, we investigated the impact of standard dementia testing scores, such as CAMCOG, when combined with the spectral qEEG data. For AD vs DLB classification, we achieved the same specificity of 0.75 but with a marked increase in sensitivity to 0.95, displaying the power of combining simple EC and EO qEEG features with standard clinical testing data to differentiate between these dementia groups.

To investigate the overall impact of the EO data on classification, we also compared the results of the three widely used classifiers: KNN, support vector machines (SVM) and logistic regression (LR). It was found that all three machine learning methods performed better when identifying individuals with Lewy body disease with the inclusion of EO qEEG features; these results are summarised in Table 1.

Additionally, we have also investigated the capability of EO data to improve the classification accuracies of a multi-class k-nearest neighbour machine learning approach. Here, healthy individuals are first filtered out from possible non-healthy participants with a second stage to identify a specific dementia group against other individuals determined to be non-healthy participants. This is done to identify each group within the context of the whole cohort and follows the likely path that would be taken within a clinical setting. These results are summarised in Table 4. In the case of identifying HCs, the classifier using the additional EO data achieved an overall accuracy of 87.70% ± 1.34 with a sensitivity and specificity of 93.3% ±1.7 and 86.0% ±7.3, respectively. These results are higher than that achieved by the EC only classifier which achieved an overall accuracy of 84.30% ±2.29 with a reduced specificity and sensitivity of 86.0% ± 5.57 and 73.00% ± 2.00, respectively, thus indicating that the addition of EO data improves the ability of the model to correctly identify healthy individuals. For dementia participants, the reduction in accuracy when only utilising the EC data set is even larger. For example, a 16.7% reduction in sensitivity is observed for the classification of AD individuals against all other participants determined to be non-healthy when not using the additional EO data set. Such a substantial reduction indicates that the addition of EO features can greatly improve the model’s ability to correctly identify AD participants against other individuals. Similarly, when differentiating individuals with DLB from all other participants classified as non-healthy, we see a reduction of 9.48% in sensitivity and 7.43% for specificity, indicating that the addition of EO data provides a significant increase to the multi-class model. The only group which saw no significant change in classification results was that of the PDD group achieving similar overall classification accuracies for both classifier types.

Overall, these results indicate that EO qEEG data represents an underutilised source of data for assisting in the diagnosis and classification of dementia. The general ease of accessibility of EO EEG data, due to its common acquisition alongside standard EC data, means that it would not be difficult improving the differentiation between AD and LBD dementia types in clinical settings. It is also pertinent to consider that standard EEG setups are already widely available in many clinical settings for assisting in the diagnosis of other neurological diseases with minimum alterations required for translation to these methodologies. However, a larger validation cohort is required to further cement the strength by which EO features improve inter-group classification accuracies.

Further research into the utilisation of EC and EO qEEG for assisting in the diagnosis of dementia should likely investigate the combination of network-derived functional connectivity methods or hierarchical clustering methods [37], as these methods have already been shown excellent ability to differentiate between dementia groups. EC data has already been utilised in studies for comparing derived and weighted network measures which show a significant difference between AD and LBD groups [13, 14, 33]; thus, investigating EO-derived connectivity measures could further improve such inter-group classification methods. Moreover, the inclusion of structural information (MRI and DTI) is likely to provide additional valuable information to further improve diagnostic accuracy. A lack of research studies utilising EO data may be driven by the need for highly trained technical staff to identify and clean EEG signals. EC EEG is certainly easier to pre-process than EO since, by definition, EC segments have fewer ocular movement artefacts and no blink artefacts. We placed special care on correctly cleaning the more challenging EO segments while following the same cleaning protocols for both EO and EC recordings, to decrease any bias that may have been produced by the cleaning procedure.

Another major benefit of utilising qEEG features is the ease of integration into new technologies. For example, deep convolutional neural networks have been shown to be an excellent method for identifying disease types in both medical imaging tasks [38, 39] and classifying EEG signals from epileptic patients [40] in addition to dementia patients from healthy individuals [41]. Deep neural networks and other similar methodologies could easily integrate EC and EO qEEG features presented within this study in future, to further strengthen classification results. More complex methodologies, such as deep neural networks, were not utilised in this study due to the need for extensive datasets. Additionally, we chose to focus on qEEG features as these are already established in clinical practice, which will likely speed up its integration into clinical environments.

## Limitations

While our work constitutes a proof-of-principle, future work leveraging data from larger independent participant cohorts will be required for two major reasons. Firstly, replication is an important part of validating scientific studies; however, we do not have access to a large enough independent cohort to this end. Secondly, a larger dataset will ensure that measurements are not biased or overfitted to any group of patients.

## Conclusions

Our study has found that EO qEEG data contains several features with significant inter-group variability for the classification between healthy controls and dementia groups, in addition to dementia inter-group analysis. By including such EO qEEG features, we achieved similar classification results to previous qEEG studies using more complex computational methods. Overall, the combination of EC and EO qEEG features improves the overall classification accuracy for HC-D and AD-DLB.

Additionally, we found a yet undocumented phenomenon between healthy controls and dementia patients when one compares the difference in DFV across brain regions in the EC and EO states. Healthy controls displayed a significantly higher DFV in the EO state, which was not seen in the dementia patients. This is possibly representing an underlying biomarker for inter-group classification; this should be further investigated with functional connectivity methods.

Overall, our study presents EO qEEG as an underutilised significant dataset for assisting in the diagnosis of dementia patients in combination with EC data, improving inter-group classification accuracy for classifying between HC-D and AD-DLB datasets. Additionally, we present undocumented differences between the HC and dementia groups which are not seen while utilising only the EC dataset.

## Supplementary Information

**Additional file 1: Supplementary Table 1.** All Abbreviations and their full names from throughout the paper. **Supplementary Table 2.** Demographic and clinical variables for HC, AD, DLB and PDD groups, including descriptive statistics for each variable. **Supplementary Table 3.** Outputs from one way four group ANOVA, with post-hoc unpaired Bonferroni correction. For testing the significance of the difference between dementia patient’s MMSE, CAMCOG and NPI hal values. With a significant difference seen in AD patients CAMCOG memory and NPI hal scores when compared to DLB and PDD patients. Additionally, a significant difference is seen between AD and DLB patients for CAMCOG total that is not seen when comparing AD and PDD patients. **Supplementary Table 4.** Outputs from unpaired t-test between each dementia subgroup for cholinesterase inhibitor usage. With no significant inter-group difference (*p*-value < 0.05) for any two subgroup comparisons. **Supplementary Table 5.** Outputs from one way four group ANOVA, with post-hoc unpaired Bonferroni correction. For testing the significance of the difference between HC and dementia patient’s theta-alpha ratio (TAR) and dominant frequency (DF) in the parietal and occipital regions. With a significant decrease in the DF of dementia patients not only in the EC but also the EO resting state. In addition, the TAR was found to also be significantly different for the DLB and PDD groups when compared to healthy controls in the same regions. **Supplementary Table 6.** Outputs from one-way ANOVA, four group, with post-hoc unpaired Bonferroni correction. For testing the significance of change in DFV between the EO and EC resting state for HC, AD, DLB and PDD patients. Notably, HC was found to be the only group to experience a significant change between the two states when compared to other groups. In addition, no dementia group was found to have a significant difference between the two states when compared with other dementia groups. **Supplementary Figure 1.** Figures showing the total number of times that full feature set for HC-D (A) classification were selected. Utilising training and testing data sets across 100 simulated runs. With features consisting of the relative delta, theta, high theta, alpha and delta power in addition to the ration of the high theta-alpha relative power (TAR) dominant frequency (DF), dominant frequency variance (DFV) and the ratio of the dominant frequency variance between the EC and EO state (EC/EO). **Supplementary Figure 2.** EEG data scrolls in the EC and EO state exported from EEGLAB for examples of AD, DLB and PDD patients with an exemplary HC example for displaying DFV differences between both stats. Firstly, this Figure displays the expected alpha rhythms (arrow) in the EC state for the HC participant which are not present for the AD, DLB or PDD participants. Secondly, displaying the difference between the EC and EO state for all participants with a notable decrease in wavelength for the HC participant within the EO state when compared to the EC with the loss of the alpha rhythms. It is notable that no significant difference is seen between the EC and EO state for any dementia patient.

## Data Availability

The dataset used and analysed during this study in addition to the relevant code are available from J.J. upon reasonable request.
