# Supplementary material for: Investigating the power of eyes open resting state EEG for assisting in dementia diagnosis
Source: Alzheimers Res Ther. 2022 Aug 5;14:109. doi: 10.1186/s13195-022-01046-z (PMC9354304; doi:10.1186/s13195-022-01046-z)
Supplement: Supplementary file 1 — Additional file 1: Supplementary Table 1. All Abbreviations and their full names from throughout the paper. Supplementary Table 2. Demographic and clinical variables for HC, AD, DLB and PDD groups, including descriptive statistics for each variable. Supplementary Table 3. Outputs from one way four group ANOVA, with post-hoc unpaired Bonferroni correction. For testing the significance of the difference between dementia patient’s MMSE, CAMCOG and NPI hal values. With a significant difference seen in AD patients CAMCOG memory and NPI hal scores when compared to DLB and PDD patients. Additionally, a significant difference is seen between AD and DLB patients for CAMCOG total that is not seen when comparing AD and PDD patients. Supplementary Table 4. Outputs from unpaired t-test between each dementia subgroup for cholinesterase inhibitor usage. With no significant inter-group difference (p-value < 0.05) for any two subgroup comparisons. Supplementary Table 5. Outputs from one way four group ANOVA, with post-hoc unpaired Bonferroni correction. For testing the significance of the difference between HC and dementia patient’s theta-alpha ratio (TAR) and dominant frequency (DF) in the parietal and occipital regions. With a significant decrease in the DF of dementia patients not only in the EC but also the EO resting state. In addition, the TAR was found to also be significantly different for the DLB and PDD groups when compared to healthy controls in the same regions. Supplementary Table 6. Outputs from one-way ANOVA, four group, with post-hoc unpaired Bonferroni correction. For testing the significance of change in DFV between the EO and EC resting state for HC, AD, DLB and PDD patients. Notably, HC was found to be the only group to experience a significant change between the two states when compared to other groups. In addition, no dementia group was found to have a significant difference between the two states when compared with other dementia groups. Supplementary Figure 1 [file 13195_2022_1046_MOESM1_ESM.zip › Supplementary material.pdf]

	HC (N = 15)	AD (N = 12)	DLB (N =21)	PDD (N = 17)	p-value
Age	76.93 ± 4.57	74.42 ± 8.97	75.81 ± 6.77	73.88 ± 5.01	F(3,64) = 0.725, p-value = 0.541[T]
Male/Female	8 \ 7	8 \ 4	18 \ 3	15 \ 2	X^2 (3, N = 65) = 22.71 , p-value = 0.418 [ǂ]
MMSE	29.13 ± 0.83	20.08 ± 4.94	23.33 ± 4.35	23.18 ± 4.76	F(2,49) = 2.15, p-value = 0.128*
CAMCOG total	96.93 ± 3.67	64.50 ± 21.41	78.57 ± 12.31	74.94 ± 13.47	F(2,49) = 3.291, p-value = 0.046*
CAMCOG executive	22.40 ± 2.10	14.67 ± 6.67	13.90 ± 5.08	12.65 ± 2.87	F(2,49) = 0.638, p-value = 0.533*
CAMCOG memory	23.87 ± 1.18	10.50 ± 5.95	18.67 ± 4.81	17.82 ± 5.08	F(2,49) = 10.41, p-value < 0.001*
CAMCOG attention	6.80 ± 0.56	3.67 ± 2.67	0.67 ± 0.48	0.88 ± 0.33	F(2,49) = 1.68, p-value = 0.198*
NPI hall	0.00 ± 0	0.00 ± 0	0.67 ± 0.48	0.88 ± 0.33	F(2,49) = 7.94, p-value = 0.001*
CAF total	0.00 ± 0	0.64 ± 1.43	3.52 ± 3.95	6.38 ± 4.46	t(36) = 1.95, p-value = 0.058[Ŧ]
Achel (yes/no)	0 \ 15	11 \ 1	19 \ 2	13 \ 4[†]	X^2 (3, N = 50) = 2.91 , p-value = 0.573
Years since diagnosis	0 ± 0	1.96 ± 1.23	0.90 ± 0.63	1.47 ± 2.07	H(2) = 9.33, p-value = 0.009[≠]

[T] Four group ANNOVA
[*] Three group ANNOVA (AD, DLB, PDD)
[ǂ] X^2 test four groups
[Ŧ] Unpaired t-test (DLB vs PDD)
[≠] Kruskal-Wallis three groups (AD, DLB, PDD)
ł One PDD patient was on Memantine

Supplementary Table 2

Demographic and clinical variables for HC, AD, DLB and PDD groups, including descriptive statistics for each variable.

Bonferroni					
Dependent Variable			Mean Difference (I-J)	Std. Error	Sig.
MMSE	Alzheimers	Lewy Body	-3.250	1.678	0.176
		PDD	-3.093	1.748	0.250
	Lewy Body	Alzheimers	3.250	1.678	0.176
		PDD	0.157	1.512	1.000
	PDD	Alzheimers	3.093	1.748	0.250
		Lewy Body	-0.157	1.512	1.000
CAMCOG attention	Alzheimers	Lewy Body	-1.333	0.736	0.230
		PDD	-0.686	0.767	1.000
	Lewy Body	Alzheimers	1.333	0.736	0.230
		PDD	0.647	0.664	1.000
	PDD	Alzheimers	0.686	0.767	1.000
		Lewy Body	-0.647	0.664	1.000
CAMCOG memory	Alzheimers	Lewy Body	-8.167*	1.877	0.000
		PDD	-7.324*	1.955	0.001
	Lewy Body	Alzheimers	8.167*	1.877	0.000
		PDD	0.843	1.692	1.000
	PDD	Alzheimers	7.324*	1.955	0.001
		Lewy Body	-0.843	1.692	1.000
CAMCOG total	Alzheimers	Lewy Body	-14.071*	5.529	0.043
		PDD	-10.441	5.761	0.229
	Lewy Body	Alzheimers	14.071*	5.529	0.043
		PDD	3.630	4.985	1.000
	PDD	Alzheimers	10.441	5.761	0.229
		Lewy Body	-3.630	4.985	1.000
CAMCOG executive	Alzheimers	Lewy Body	0.762	1.779	1.000
		PDD	2.020	1.854	0.845
	Lewy Body	Alzheimers	-0.762	1.779	1.000
		PDD	1.258	1.604	1.000
	PDD	Alzheimers	-2.020	1.854	0.845
		Lewy Body	-1.258	1.604	1.000
NPI hal	Alzheimers	Lewy Body	-.667*	0.139	0.000
		PDD	-.882*	0.145	0.000
	Lewy Body	Alzheimers	.667*	0.139	0.000
		PDD	-0.216	0.122	0.251
	PDD	Alzheimers	.882*	0.145	0.000
		Lewy Body	0.216	0.122	0.251

*. The mean difference is significant at the 0.05 level.

Supplementary Table 3

Outputs from one way four group ANOVA, with post-hoc unpaired Bonferroni correction. For testing the significance of the difference between dementia patient's MMSE, CAMCOG and NPI hal values. With a significant difference seen in AD patients CAMCOG memory and NPI hal scores when compared to DLB and PDD patients. Additionally, a significant difference is seen between AD and DLB patients for CAMCOG total that is not seen when comparing AD and PDD patients.

	Comparison	p-value (Unpaired t-test)
Achel (yes/no)	AD-DLB	t(31) = 0.11, p-value = 0.912
	AD-PDD	t(27) = 1.13, p-value = 0.268
	DLB-PDD	t(36) = 1.36, p-value = 0.174

Supplementary Table 4

Outputs from unpaired t-test between each dementia subgroup for cholinesterase inhibitor usage. With no significant inter-group difference (p-value < 0.05) for any two subgroup comparisons.

Multiple Comparisons					
Dependent Variable			Mean Difference (I-J)	Std. Error	Sig.
EC Parietal TAR	HC	AD	-.685474323000*	0.205073176338	0.009
		DLB	-1.160637475000*	0.179002543538	0.000
		PDD	-1.418087917000*	0.187572051160	0.000
EC Parietal DF		AD	1.411996647000*	0.330132361482	0.000
		DLB	2.034666058000*	0.288163149685	0.000
		PDD	2.398356530000*	0.301958575487	0.000
EC Occipital TAR		AD	-0.994285423133	0.560412003571	0.486
		DLB	-2.079905764000*	0.489167700328	0.000
		PDD	-2.900918470000*	0.512585950449	0.000
EC Occipital DF		AD	1.431841761000*	0.366136572680	0.001
		DLB	1.947901017000*	0.319590201714	0.000
		PDD	2.473971039000*	0.334890155645	0.000
EO Parietal TAR		AD	-0.344958658167	0.174316047054	0.314
		DLB	-.784353611000*	0.152155520089	0.000
		PDD	-.965251753000*	0.159439762332	0.000
EO Parietal DF		AD	.970469026000*	0.353936892004	0.048
		DLB	1.538216722000*	0.308941447399	0.000
		PDD	1.797302838000*	0.323731606445	0.000
EO Occipital TAR		AD	-0.257644112883	0.115941622996	0.180
		DLB	-.507921364000*	0.101202145443	0.000
		PDD	-.658223746000*	0.106047062949	0.000
EO Occipital DF		AD	.919914401000*	0.319690445096	0.033
		DLB	1.347306948000*	0.279048697830	0.000
		PDD	1.594761672000*	0.292407781427	0.000

*. The mean difference is significant at the 0.05 level.

Supplementary Table 5

Outputs from one way four group ANOVA, with post-hoc unpaired Bonferroni correction. For testing the significance of the difference between HC and dementia patient's theta-alpha ratio (TAR) and dominant frequency (DF) in the parietal and occipital regions. With a significant decrease in the DF of dementia patients not only in the EC but also the EO resting state. In addition, the TAR was found to also be significantly different for the DLB and PDD groups when compared to healthy controls in the same regions.

Multiple Comparisons

Dependent Variable			Mean Difference (I-J)	Std. Error	Sig.	Dependent Variable			Mean Difference (I-J)	Std. Error	Sig.
Frontal	HC	AD	-.250309332000*	0.073016469974	0.007	Parietal	HC	AD	-.340715305000*	0.073736590935	0.000
		DLB	-.262308853000*	0.063734000121	0.001			DLB	-.337744177000*	0.064362573228	0.000
		PDD	-.283416886000*	0.066785180227	0.000			PDD	-.376593472000*	0.067443845432	0.000
	AD	HC	.250309332000*	0.073016469974	0.007		AD	HC	.340715305000*	0.073736590935	0.000
		DLB	-0.011999520869	0.068223171226	1.000			DLB	0.002971127619	0.068896018539	1.000
		PDD	-0.033107553936	0.071081914850	1.000			PDD	-0.035878167000	0.071782956367	1.000
	DLB	HC	.262308853000*	0.063734000121	0.001		DLB	HC	.337744177000*	0.064362573228	0.000
		AD	0.011999520869	0.068223171226	1.000			AD	-0.002971127619	0.068896018539	1.000
		PDD	-0.021108033067	0.061508182405	1.000			PDD	-0.038849294619	0.062114803506	1.000
	PDD	HC	.283416886000*	0.066785180227	0.000		PDD	HC	.376593472000*	0.067443845432	0.000
		AD	0.033107553936	0.071081914850	1.000			AD	0.035878167000	0.071782956367	1.000
		DLB	0.021108033067	0.061508182405	1.000			DLB	0.038849294619	0.062114803506	1.000
Central	HC	AD	-.269057862000*	0.065075221277	0.001	Occipital	HC	AD	-.287710740000*	0.097697171445	0.027
		DLB	-.255124628000*	0.056802309975	0.000			DLB	-.373062061000*	0.085277082539	0.000
		PDD	-.290136173000*	0.059521644677	0.000			PDD	-.351951385000*	0.089359608935	0.001
	AD	HC	.269057862000*	0.065075221277	0.001		AD	HC	.287710740000*	0.097697171445	0.027
		DLB	0.013933233845	0.060803240219	1.000			DLB	-0.085351320631	0.091283663237	1.000
		PDD	-0.021078311446	0.063351067771	1.000			PDD	-0.064240645387	0.095108706629	1.000
	DLB	HC	.255124628000*	0.056802309975	0.000		DLB	HC	.373062061000*	0.085277082539	0.000
		AD	-0.013933233845	0.060803240219	1.000			AD	0.085351320631	0.091283663237	1.000
		PDD	-0.035011545291	0.054818571506	1.000			PDD	0.021110675244	0.082298903848	1.000
	PDD	HC	.290136173000*	0.059521644677	0.000		PDD	HC	.351951385000*	0.089359608935	0.001
		AD	0.021078311446	0.063351067771	1.000			AD	0.064240645387	0.095108706629	1.000
		DLB	0.035011545291	0.054818571506	1.000			DLB	-0.021110675244	0.082298903848	1.000
Temporal	HC	AD	-.219244977000*	0.066750429167	0.010	*. The mean difference is significant at the 0.05 level.					
		DLB	-.261402110000*	0.058264551301	0.000						
		PDD	-.307833222000*	0.061053888852	0.000						
	AD	HC	.219244977000*	0.066750429167	0.010						
		DLB	-0.042157133250	0.062368476046	1.000						
		PDD	-0.088588244779	0.064981891401	1.000						
	DLB	HC	.261402110000*	0.058264551301	0.000						
		AD	0.042157133250	0.062368476046	1.000						
		PDD	-0.046431111529	0.056229746170	1.000						
	PDD	HC	.307833222000*	0.061053888852	0.000						
		AD	0.088588244779	0.064981891401	1.000						
		DLB	0.046431111529	0.056229746170	1.000						

Supplementary Table 6

Outputs from one-way ANOVA, four group, with post-hoc unpaired Bonferroni correction. For testing the significance of change in DFV between the EO and EC resting state for HC, AD, DLB and PDD patients. Notably, HC was found to be the only group to experience a significant change between the two states when compared to other groups. In addition, no dementia group was found to have a significant difference between the two states when compared with other dementia groups.

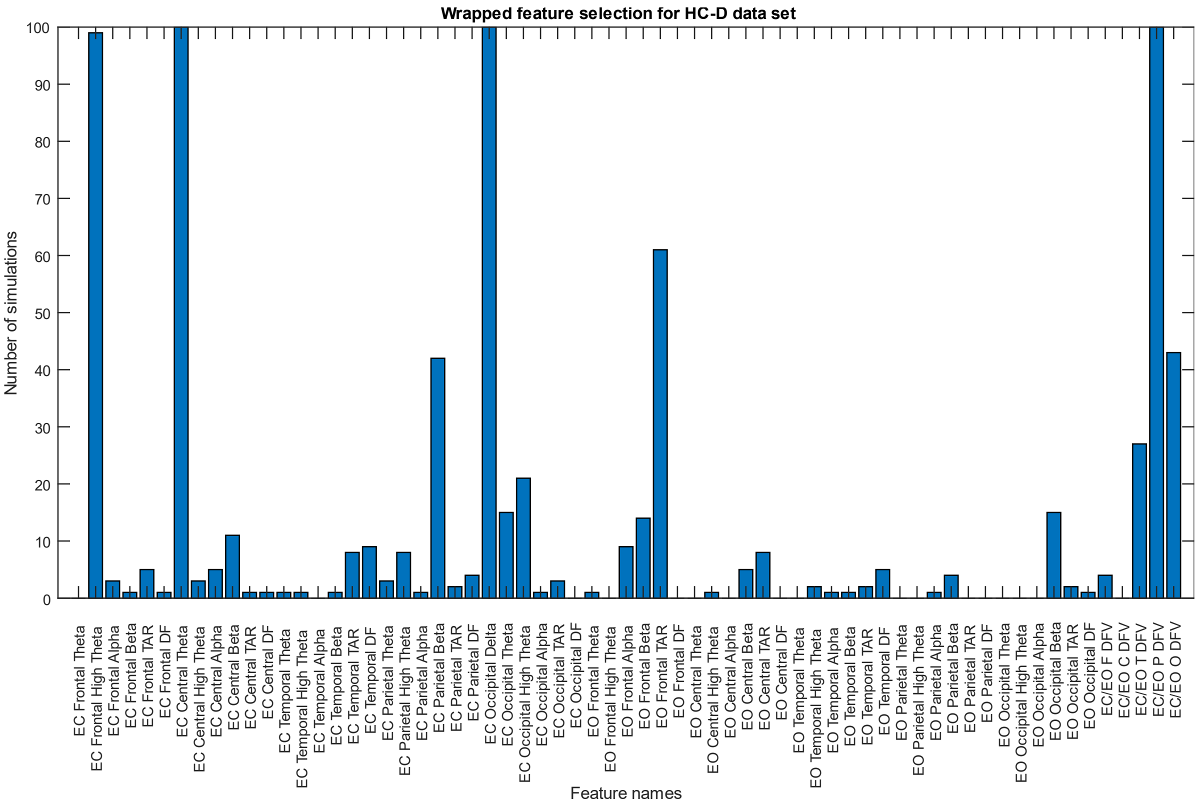

Supplementary Figure 1

Figures showing the total number of times that full feature set for HC-D (A) classification were selected. Utilising training and testing data sets across 100 simulated runs. With features consisting of the relative delta, theta, high theta, alpha and delta power in addition to the ration of the hightheta-alpha relative power (TAR) dominant frequency (DF), dominant frequency variance (DFV) and the ratio of the dominant frequency variance between the EC and EO state (EC/EO).

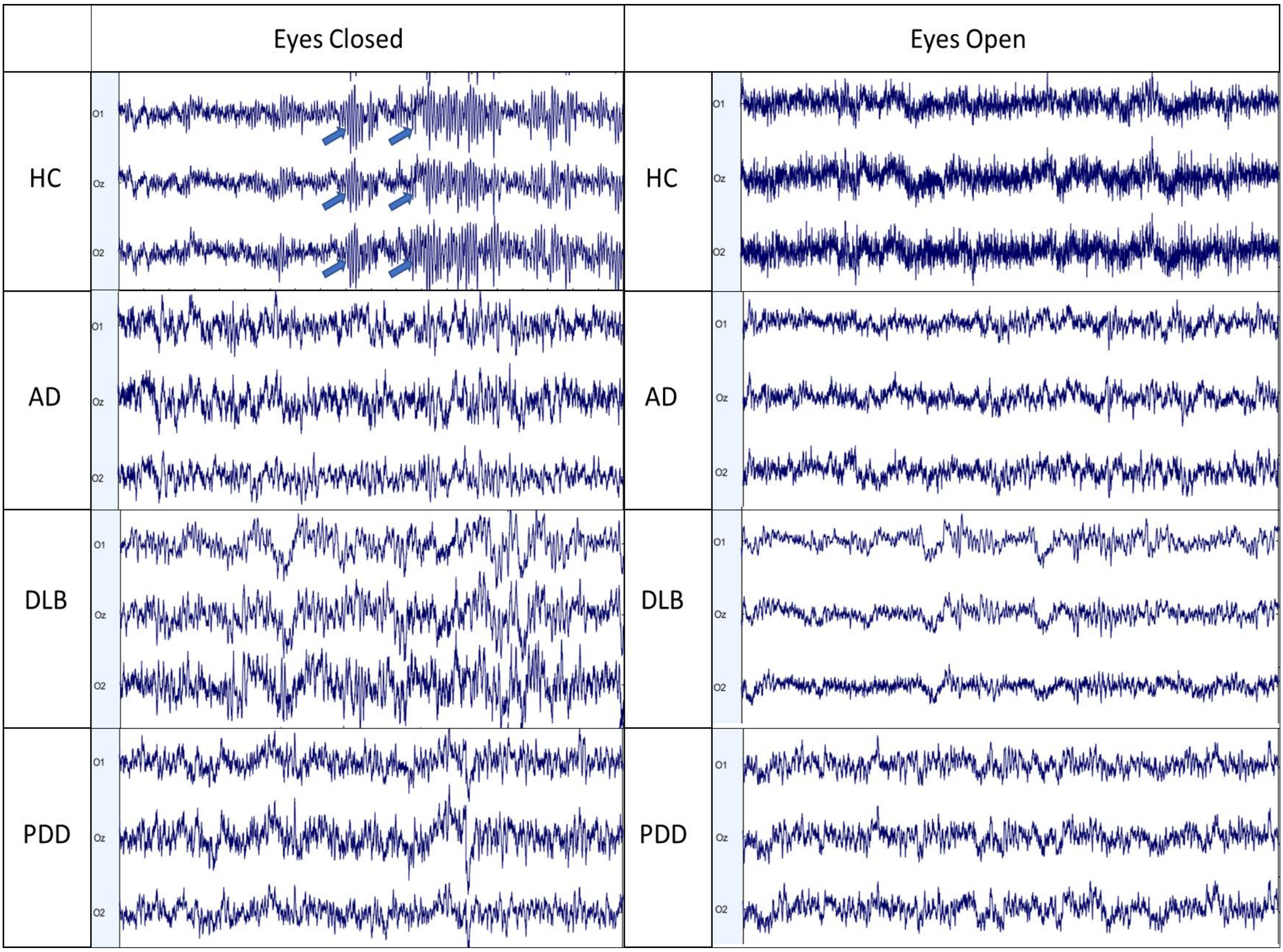

Supplementary Figure 2

EEG data scrolls in the EC and EO state exported from EEGLAB for examples of AD, DLB and PDD patients with an exemplary HC example for displaying DFV differences between both stats. Firstly, this Figure displays the expected alpha rhythms (arrow) in the EC state for the HC participant which are not present for the AD, DLB or PDD participants. Secondly, displaying the difference between the EC and EO state for all participants with a notable decrease in wavelengthfor the HC participant within the EO state when compared to the EC with the loss of the alpha rhythms. It is notable that no significant difference is seen between the EC and EO state for any dementia patient.
