# Supplementary material for: Investigating the power of eyes open resting state EEG for assisting in dementia diagnosis
Source: Alzheimers Res Ther. 2022 Aug 5;14:109. doi: 10.1186/s13195-022-01046-z (PMC9354304; doi:10.1186/s13195-022-01046-z)
Supplement: Supplementary file 1 — Additional file 1: Supplementary Table 1. All Abbreviations and their full names from throughout the paper. Supplementary Table 2. Demographic and clinical variables for HC, AD, DLB and PDD groups, including descriptive statistics for each variable. Supplementary Table 3. Outputs from one way four group ANOVA, with post-hoc unpaired Bonferroni correction. For testing the significance of the difference between dementia patient’s MMSE, CAMCOG and NPI hal values. With a significant difference seen in AD patients CAMCOG memory and NPI hal scores when compared to DLB and PDD patients. Additionally, a significant difference is seen between AD and DLB patients for CAMCOG total that is not seen when comparing AD and PDD patients. Supplementary Table 4. Outputs from unpaired t-test between each dementia subgroup for cholinesterase inhibitor usage. With no significant inter-group difference (p-value < 0.05) for any two subgroup comparisons. Supplementary Table 5. Outputs from one way four group ANOVA, with post-hoc unpaired Bonferroni correction. For testing the significance of the difference between HC and dementia patient’s theta-alpha ratio (TAR) and dominant frequency (DF) in the parietal and occipital regions. With a significant decrease in the DF of dementia patients not only in the EC but also the EO resting state. In addition, the TAR was found to also be significantly different for the DLB and PDD groups when compared to healthy controls in the same regions. Supplementary Table 6. Outputs from one-way ANOVA, four group, with post-hoc unpaired Bonferroni correction. For testing the significance of change in DFV between the EO and EC resting state for HC, AD, DLB and PDD patients. Notably, HC was found to be the only group to experience a significant change between the two states when compared to other groups. In addition, no dementia group was found to have a significant difference between the two states when compared with other dementia groups. Supplementary Figure 1 [file 13195_2022_1046_MOESM1_ESM.zip › Supplementary Table 1.docx]

**Table of abbreviations:**

Supplementary Table 1.

All Abbreviations and their full names from throughout the paper.
